# Patient‐incurred costs in a differentiated service delivery club intervention compared to standard clinical care in Northwest Tanzania

**DOI:** 10.1002/jia2.25760

**Published:** 2021-06-23

**Authors:** Nwanneka E Okere, Lucia Corball, Dunia Kereto, Sabine Hermans, Denise Naniche, Tobias F Rinke de Wit, Gabriela B Gomez

**Affiliations:** ^1^ Amsterdam Institute for Global Health and Development Department of Global Health Amsterdam UMC University of Amsterdam Amsterdam Netherlands; ^2^ Bugisi Health Centre Shinyanga Tanzania; ^3^ ISGLOBAL‐Barcelona Institute for Global Health Hospital Clinic University of Barcelona Barcelona Spain; ^4^ Department of Global Health and Development London School of Hygiene and Tropical Medicine London United Kingdom; ^5^ Present address: Vaccine epidemiology and modelling Department Sanofi Pasteur Lyon France

**Keywords:** costs, antiretroviral treatment, differentiated service delivery, Tanzania, catastrophic costs, patient‐incurred costs

## Abstract

**Introduction:**

Placing all clients with a positive diagnosis for HIV on antiretroviral therapy (ART) has cost implications both for patients and health systems, which could, in turn, affect feasibility, sustainability and uptake of new services. Patient‐incurred costs are recognized barriers to healthcare access. Differentiated service delivery (DSD) models in general and community‐based care in particular, could reduce these costs. We aimed to assess patient‐incurred costs of a community‐based DSD intervention (clubs) compared to clinic‐based care in the Shinyanga region, Tanzania.

**Methods:**

Cross‐sectional survey among stable ART patients (n = 390, clinic‐based; n = 251, club‐based). For each group, we collected socio‐demographic, income and expenditure data between May and August 2019. We estimated direct and indirect patient‐incurred costs. Direct costs included out‐of‐pocket expenditures. Indirect costs included income loss due to time spent during transport, accessing services and off work during illness. Cost drivers were assessed in multivariate regression models.

**Results:**

Overall, costs were significantly higher among clinic participants. Costs (USD) per year for clinic versus club were as follows: 11.7 versus 4.17 (*p* < 0.001) for direct costs, 20.9 versus 8.23 (*p* < 0.001) for indirect costs and 32.2 versus 12.4 (*p* < 0.001) for total costs. Time spent accessing care and time spent in illness (hours/year) were 38.3 versus 13.8 (*p* < 0.001) and 16.0 versus 6.69 (*p* < 0.001) respectively. The main cost drivers included transportation (clinic vs. club: 67.7% vs. 44.1%) for direct costs and income loss due to time spent accessing care (clinic vs. club: 60.4% vs. 56.7%) for indirect costs. Factors associated with higher total costs among patients attending clinic services were higher education level (coefficient [95% confidence interval]) 20.9 [5.47 to 36.3]) and formal employment (44.2 [20.0 to 68.5). Differences in mean total costs remained significantly higher with formal employment, rural residence, in addition to more frequent visits among clinic participants. The percentage of households classified as having had catastrophic expenditures in the last year was low but significantly higher among clinic participants (10.8% vs. 5.18%, *p* = 0.014).

**Conclusions:**

Costs incurred by patients accessing DSD in the community are significantly lower compared to those accessing standard clinic‐based care. DSD models could improve access, especially in resource‐limited settings.

## 1 Introduction

Placing all clients with a positive diagnosis for HIV on antiretroviral therapy (ART) as currently recommended by WHO has implications both for patients and health systems alike. In sub‐Saharan Africa, ART services are offered free to patients at the point of care. However, patients incur costs while accessing care. These patient‐incurred costs during care‐seeking include transportation, food, temporary caregiver costs, income loss during appointments, sometimes user fees or costs of other medications than ART. These are recognized as barriers to healthcare access [1, 2, 3]. Among people living with HIV (PLHIV), out‐of‐pocket (OOP) expenditure was demonstrated higher among those in low socio‐economic status (SES) and rural residence [4], possibly contributing to poverty due to catastrophic expenditures [1, 3, 5].

Differentiated service delivery (DSD) is a patient‐centred strategy currently promoted for HIV services [6]. DSD entails providing different options of service access to PLHIV based on their clinical status. Although largely community‐based (e.g. ART‐clubs, community ART groups), clinic‐based DSD options also exist including fast‐track refills and multi‐month scripting [7, 8, 9, 10, 11]. As DSD interventions become more widespread, it is pertinent to understand the impact on patient‐incurred costs.

In Tanzania, with a generalized HIV epidemic, DSD is recommended in the current HIV care and treatment guidelines as a patient‐centred strategy to expand access [12]. For stable patients, both facility‐based and community‐based models are recommended. Studies looking at these service delivery options showcase patient‐related benefits such as improved retention in care, viral suppression and peer support [11, 13, 14]. There is evidence of patient‐incurred cost reduction in community‐based services similar to DSD [15, 16] in addition to evidence of a reduction in costs for implementing DSD interventions compared with standard clinical care [17, 18, 19, 20] from a healthcare provider perspective. However, studies estimating the cost implications of DSD from patients' perspectives are limited [3, 21, 22].

Therefore, to fill this gap, this study aimed to determine the patient‐incurred cost incurred by stable ART patients both accessing clinic‐based care and DSD in adherence clubs in Shinyanga, a largely rural region in Tanzania.

## 2 Methods

We conducted a cross‐sectional survey nested within the Test & Treat (TnT) implementation project, details of which have been published elsewhere [23]. Briefly, clinics in two care and treatment centres (CTC) and 24 adherence clubs were set up as part of the TnT project. All sites are owned by a Catholic mission providing HIV services as well as other services free of charge such as routine laboratory monitoring tests, for example CD4 count, viral load (VL), etc., medications for common opportunistic infections, breakfast for patients during clinic appointments. VL is determined centrally by Bugando Hospital reference laboratories. Adherence clubs are affiliated to the CTC sites within nearby communities. The TnT treatment sites covered different contextual realities prevailing within the region, including widely dispersed rural and more densely populated peri‐urban areas. Care was clinic‐based in CTC sites for most patients with visits scheduled every three months (but could be more frequent depending on clinical status). Club‐based care became an option mid‐2018 for stable ART patients who chose this option and visits were scheduled every three months. One of the four meetings planned in a year is scheduled as a clinic visit for club members. These patients were free to attend the clinic whenever they fall ill between club meetings.

Stable patients were eligible to participate in our study if they were 18 years of age or above and had been accessing services at the study sites for at least one year and/or had received follow‐up care in the DSD clubs for at least six months before the beginning of the study. Stable patients were identified according to the Tanzanian HIV care and treatment guidelines as those on first‐line anti‐retrovirals for at least six months, have not had any adverse drug reactions that require regular monitoring and no current illnesses (opportunistic infections and/or comorbidities) [12]. They have demonstrated good adherence with VL below 50 copies/mL or a CD4 cell count of above 350/μL (in absence of VL) and good clinic attendance for the past six months assessed as attending all scheduled visits.

Participants were recruited among eligible patients attending clinic appointments or club meetings. In the clinics, a random list of participants was generated of all eligible patients with scheduled clinic appointments during the study period. In the clubs, all members were stable and offered participation in the study. After their consultation, those patients approached and providing informed consent in their preferred language were then asked to answer the study questionnaire. The study questionnaire was an adaptation of existing cost survey tools [24, 25]. The WHO Global TB tool provided the format for collecting socio‐demographic data, household income and information on patient‐incurred costs, whereas the HIV programme costing tool informed the cost categories. Costs data were collected at one time point during the data collection period. Participants were asked about their expenditures over the past six months to estimate direct and indirect costs. Six‐month cost estimates were multiplied by two to estimate the annual cost per participant. The survey questionnaire was piloted on 15 clients to test for clarity and refine where necessary. Trained research assistants who could speak English, Swahili and Sukuma administered the survey. We also extracted the number of visits from participants’ paper medical records and club registers. Data collection occurred within three months between May and August 2019. Double data entry was done into Epidata 4.6 software [26], data files were compared and discrepancies were checked using the paper questionnaire. Data were analysed using STATA 16.0 SE [27].

### 2.1 Data analysis

We compared the two population samples (clinic‐ and club‐based) using descriptive statistics in terms of sex, age, education, marital status, occupation, location, SES, years on ART, number of visits and insurance. To assess SES, we used the Tanzania DHC 2015 equity tool and collected data on asset ownership to calculate an asset‐based wealth index (i.e. ownership of TV, radio, available bank accounts among others) [28]. The index was obtained through principal component analysis and patients were categorized into five SES quintiles with the lowest [1] representing the poorest SES.

Patient‐incurred costs were categorized into direct and indirect costs. Direct costs included medical and non‐medical costs. Direct medical costs were derived by adding all OOP expenditures for additional medicines, laboratory tests or other consultations not provided as part of the free HIV service (especially, in‐between scheduled visits). Direct non‐medical costs included transportation costs, expenses incurred while accessing care such as food, accommodation, dietary supplements and the costs of temporary caregivers. Indirect costs included access‐ and illness‐related costs. These were estimated from the reported time spent accessing care or while unable to work multiplied by the estimated individual income. Income was derived from the questionnaire (reported monthly income) and we assumed an average of 8 hours/workday for 24 days per month to derive income per hour. Total patient‐incurred costs associated with HIV care were calculated by summing direct (medical and non‐medical) and indirect (access‐ and illness‐related) costs. Costs afforded through coping strategies such as borrowing money and selling fixed assets were reported separately to describe coping strategies available in this population. Catastrophic expenditure was defined as patient‐incurred medical costs exceeding 10% (also assessed at 5% and 20%) of reported household income [3, 29].

Predictors of costs were identified using quantile (median) regression. The final regression model included all covariates. We used the Oaxaca decomposition method to quantify the difference in total costs between clinic and club attributable to participant characteristics [30]. The decompose package available in STATA was adapted to perform median regression. Costs are reported in United States dollars (USD) for 2019, using average conversion rates for the data collection period (2305.53 Tanzanian Shillings (TZS) to 1 USD) [31].

## 3 Results

Of a total of 667 clients invited, 641 consented to participate (96%). The most frequent reason for non‐participation was the lack of time for an interview due to other scheduled engagements. Characteristics of participants are presented in Table 1 by the ART service site. The majority of participants were female (63.2%) in both settings, although the proportion of male participants was higher in clinics than in clubs (40.3% vs. 31.5%). The median age was higher among club participants than clinic (39.9 vs. 44.7 years) with nearly half of all club participants aged 45 years or more. Education level was low among all participants: about a quarter had received no formal education and 69.7% attained primary level only. Over 80% of all participants were either married (52.7%) or had once been married (30.6%). Subsistence farming was the predominant occupation across settings. There were more rural dwellers among all participants, with significantly more attending clinics compared to the clubs. Both personal ($79.4 vs. 61.9) and household ($89.2 vs. 68.0) mean monthly income, as well as the asset index, were similarly distributed in the clinic and club settings.

**Table 1:** Characteristics of study participants

Variables	Clinic (N = 390)	Club (N = 251)
Sex (n, %)		
Female	233, 59.7	172, 68.3
Male	157, 40.3	79, 31.5
Age median (IQR)	39.9 (33.6 to 48.9)	44.7 (37.6 to 54.0)
18 to 25 (n, %)	25, 6.4	6, 2.4
25 to 35	97, 24.9	35, 13.9
35 to 45	138, 35.4	91, 36.2
45 to 55	80, 20.5	62, 24.7
55 to 65	35, 8.9	40, 15.9
>65	15, 3.8	17, 6.8
Education (n, %)		
None	99, 25.4	60, 23.9
Primary	267, 68.5	180, 71.7
≥Secondary	24, 6.15	11, 4.4
Marital status (n, %)		
Single	64, 16.4	43, 17.1
Married	218, 55.9	120, 47.8
Separated/divorced/widow	108, 27.7	88, 35.1
Occupation (n, %)		
Farming	213, 54.6	85, 33.9
Small‐scale business	80, 20.5	72, 28.7
Daily labour	34, 8.72	31, 12.3
Paid job	6, 1.54	2, 0.8
Unemployed	57, 14.6	61, 24.3
Location		
Rural	334, 85.6	168, 66.9
Urban	56, 14.4	83, 33.1
Mean monthly income mean (SD)		
Personal	79.4 (193.2)	61.9 (81.9)
Household	89.2 (200.2)	68.0 (83.4)
Socio‐economic status^a^ (asset index quintile) n, %		
1 (lowest)	107, 27.5	66, 26.4
2	85, 21.8	48, 19.2
3	55, 14.1	27, 10.8
4	69, 17.7	55, 22.0
5 (highest)	73, 18.8	54, 21.6
Years on ART		
≤1 year	23, 5.90	4, 1.59
1 to 5 years	225, 57.7	135, 53.2
>5 years	133, 34.1	105, 41.8
Missing	9, 2.31	7, 2.79
Number of visits		
4	25, 6.41	130, 51.8
6	251, 64.4	95, 37.8
12	114, 29.2	26, 10.4
Insurance		
Yes	31, 7.95	21, 8.37
No	358, 91.8	229, 91.2
Missing	1, 0.26	1, 0.40

^a^ Missing data for one participant each in clinic and club for SES variable.

### 3.1 Patient‐incurred costs

All costs incurred by patients per visit/per year are presented in Table 2. Overall, total costs were substantially lower among participants in clubs than in clinic settings. Indirect costs per year ($20.9 of 32.2 in clinics and $8.23 of 12.4 in clubs) contributed to the majority of total costs.

**Table 2:** Patient‐incurred costs per visit and year, 2019 USD

	Clinic (N = 390)	Club (N = 251)	Difference in means
Direct medical costs, mean (SD)			
Extra medicine cost			
Per visit	0.08 (0.97)	0.02 (0.23)	0.06 [−0.06 to 0.19]
Per year	0.72 (9.88)	0.08 (0.94)	0.64 [−0.58 to 1.87]
Out‐of‐clinic care cost			
Per month	0.19 (0.93)	0.18 (0.82)	0.01 [−0.13 to 0.15]
Per year	2.33 (11.2)	2.20 (9.82)	0.13 [−1.57 to 1.82]
Total, direct medical cost			
Per month	0.25 (1.25)	0.19 (0.84)	0.06 [−0.11 to 0.24]
Per year	3.05 (15.1)	2.27 (10.1)	0.77 [−1.35 to 2.89]
Direct non‐medical costs, mean (SD)			
Transport costs			
Per visit	1.09 (1.72)	0.30 (0.73)	0.78 [0.56 to 1.00]***
Per year	7.92 (12.7)	1.84 (4.45)	6.07 [4.43 to 7.71]***
Food costs			
Per visit	0.02 (0.13)	0.00 (0.04)	0.01 [−0.00 to 0.03]
Per year	0.15 (1.14)	0.02 (0.17)	0.14 [−0.00 to 0.28]
Accommodation costs			
Per visit	0.01 (0.11)	0.00 (0.03)	0.00 [−0.01 to 0.02]
Per year	0.07 (1.32)	0.01 (0.11)	0.06 [−0.10 to 0.22]
Temporary caregiver costs			
Per visit	0.08 (0.81)	0.01 (0.09)	0.07 [−0.03 to 0.17]
Per year	0.53 (4.92)	0.03 (0.34)	0.50 [−0.11 to 1.11]
Total, direct non‐medical costs			
Per visit	1.15 (1.93)	0.31 (0.76)	0.84 [0.59 to 1.09]***
Per year	8.61 (14.0)	1.89 (4.52)	6.71 [4.92 to 8.50]***
Total, direct costs (medical and non‐medical)			
Per visit	1.27 (2.31)	0.32 (0.78)	0.95 [0.65 to 1.25]***
Per year	11.7 (21.0)	4.17 (10.8)	7.49 [4.67 to 10.30]***
Indirect access costs, mean (SD)			
Travel time, hours			
Per visit	2.81 (1.98)	1.14 (2.68)	1.66 [1.30 to 2.03]***
Per year	20.6 (17.1)	6.25 (16.0)	14.4 [11.7 to 17.1]***
Time in clinic/club, hours			
Per visit	2.32 (1.38)	1.39 (1.37)	0.93 [0.71 to 1.15]***
Per Year	17.6 (13.3)	7.48 (8.15)	10.1 [8.23 to 11.9]***
Total time, hours			
Per visit	5.13 (2.67)	2.54 (3.03)	2.59 [2.14 to 3.04]***
Per year	38.3 (25.1)	13.8 (18.3)	24.5 [20.9 to 28.1]***
Total, indirect access costs			
Per visit	1.68 (2.57)	0.81 (1.43)	0.87 [0.51 to 1.23]***
Per year	12.2 (17.8)	4.37 (7.45)	7.81 [5.41 to 10.2]***
Indirect illness costs, mean (SD)			
Time spent in illness, hours			
Per year	16.0 (137.6)	6.69 (45.7)	9.31 [−8.34 to 26.9]
Total, indirect illness costs			
Per year	3.79 (25.9)	0.97 (8.44)	2.82 [−0.50 to 6.15]
Total, indirect costs			
Per year (only access)	12.2 (17.8)	4.37 (7.45)	7.81 [5.41 to 10.2]***
Per year (access and illness)	16.1 (31.8)	5.39 (11.4)	10.7 [6.47 to 14.9]***
Per year (access and illness and coping)	20.9 (36.7)	8.23 (20.1)	12.6 [7.53 to 17.8]***
Total costs (direct and indirect)			
Per visit	2.89 (3.63)	1.11 (1.72)	1.78 [1.29 to 2.28]
Per year	32.2 (45.7)	12.4 (25.2)	19.7 [13.4 to 26.1]***

*** *p* < 0.001.

On average, direct medical costs incurred by participants per year did not differ significantly between the two settings ($3.05 in clinics and $2.27 in clubs, *p* = 0.84). Most of the costs (82.9%) were incurred while seeking care from providers outside the CTCs involved in the TnT project. Direct non‐medical costs contributed the most (73.6% and 45.3%) to total direct costs with travel costs ($20.6 and 8.91 in clinics and clubs respectively) being significantly higher among clinic participants (*p* < 0.001). Expenditure on food and temporary caregiver was reported rarely among participants, whereas accommodation cost was reported by one clinic participant alone.

Indirect costs per year were significantly higher among clinic participants ($20.9 vs. 8.23, *p* = 0.001). The main contributor to indirect costs was income loss, that is income foregone while seeking care. Only 5% among unemployed participants would have been engaged in leisure activities alone if they had no clinic visit/club meeting. On average, the time spent accessing care, that is traveling, and waiting to receive service, was significantly higher for clinic patients. The mean total travel time spent to service location per year was three times longer for clinic participants (20.6 vs. 6.25 hours, *p* < 0.001). Similarly, the mean total waiting time was twice as long (17.6 vs. 7.48 hours, *p* < 0.001) in these settings. Likewise, the mean total time spent in ill‐health per year was considerably higher among clinic participants though not significant (16.0 vs. 6.69 hours, *p* = 0.74). This is partially a reflection of different health service utilization patterns (see Table 3) and other characteristics, for example occupation and residence. The average number of visits of any kind per year (7.9 vs. 5.8, *p* < 0.001) and associated costs were larger among clinic participants. Most patients attended clinics or clubs on scheduled visits (>91%). However, there were more patients on scheduled visits (91.3 vs. 96.0%, *p* = 0.04) and fewer missed appointments (13.4% vs. 3.59%, *p* < 0.001) among club participants compared to clinic participants.

**Table 3:** Health service utilization by service delivery model

Variables, n (%)	Clinic (N = 390)	Club (N = 251)	Difference in means
Number of visits per year mean (SD)			
Clinic visits	7.99 (3.07)	2.84 (2.59)	2.15 [ 1.69 to 2.61]***
Club meetings	‐	3	
Distribution of number of visits^a^			
Patients attending 4 visits or less	25, 6.41	130, 51.8	174.8***
Patients attending 5 to 6 visits	251, 64.4	95, 37.8	
Patients attending 7 to 12 visits	114, 29.2	26, 10.4	
Visit type^b^			
Scheduled	356, 91.3	241, 96.0	2.331**
Unscheduled	30, 7.7	10, 4.0	
Treatment supporter	4, 1.03		
Sex – number of visits mean (SD)			
Female	8.14 (3.20)	5.86 (2.51)	2.28 [1.70 to 2.85]***
Male	7.78 (2.86)	5.80 (2.78)	1.99 [1.21 to 2.76]***
Income level^c^ – number of visits mean (SD)			
<100,000	7.71 (2.96)	5.54 (2.44)	2.17 [1.60 to 2.74]***
>100,000 to 300,000	8.72 (3.24)	6.10 (2.81)	2.62 [1.65 to 3.60]***
>300,000	7.69 (2.95)	6.61 (2.68)	1.08 [−0.12 to 2.27]
Location – number of visits mean (SD)			
Rural	7.72 (2.88)	4.89 (1.79)	2.83 [2.35 to 3.31]***
Urban	9.60 (3.61)	7.76 (2.90)	1.85 [0.75 to 2.94]**
Missed visits in last 6 months^a^ (Yes/No) n, %	52, 13.4	9, 3.59	16.9***
Frequency^b^			
At least Once	44, 84.6	8, 88.9	1
>Once	8, 15.4	1, 11.1	
Reasons for missed visit^b^ n, %			
Forgot	16, 30.8	3, 33.3	0
Sick	11, 21.1	1, 11.1	
Traveled	11, 21.2	3, 33.3	
No transport fees	3, 5.77	1, 11.1	
Others	9, 21.2	1, 11.1	

^a^ Pearson’s chi2

^b^ Mann Whitney z‐score/Kruskal Wallis

^c^ <100,000TZS = $43.1; 100,000 to 300,000 = $43.1 to 130.1; >300,000 = >$130.1

* *p* < 0.05

** <0.01

*** <0.001.

Given the differences observed among participants, we analysed the cost differentials (direct, indirect and total costs) by service delivery models controlling for all covariates – see Tables S1 and S2. Across groups, the difference in direct costs remained higher among those with higher education. Differences in indirect costs remained higher among those with more frequent clinic visits. Finally, the differences in total costs were also higher in clinics among those having formal employment, rural residence and more frequent visits. The Oaxaca decomposition revealed that the differences observed in participant characteristics across groups explain only a small portion (4.8%) of the difference observed in total costs between clinic and club (Table S3).

The proportion of participants that were primary income earners per household, their average income or the average household income were similar across settings. Opportunity costs due to care access were, however, significantly higher among clinic participants. Catastrophic expenditure was significantly higher among clinic participants compared to club patients (see Table S4).

Participants reported ways in which their lives were impacted by their illness (see Table 4) which included work discontinuation either by own decision or dismissal. Disruption of social life was also reported including divorce, isolation from friends, and disruption of sex life, and was not different between clinic and club (*p* = 0.861) participants. Coping strategies implemented by participants and their households to deal with medical expenditure and income loss were similar between groups. The majority of participants did not have any insurance in clinics and clubs (7.95% vs. 8.37%) and among those who did the most common form was the National Health Insurance (61.3% vs. 71.4%). Other ways participants coped include borrowing, mostly from individuals (10.3% vs. 7.57%) and the sale of property (10.0% vs. 7.2%). We observed no difference in these regard between both study groups.

**Table 4:** Impact of HIV and coping strategies

Impact (n, %)	Clinic (N = 390)	Club (N = 251)	Z scores
Ever stopped work due to illness^a^	34, 8.7	9, 3.6	6.428*
Dismissed from work due to illness	6, 1.54	3, 1.2	1
Effect on social life^a^			
Divorce/separation from spouse	54, 13.8	31, 12.3	0.861
Isolation by friends	24, 6.1	21, 8.4	
Disruption of sex life	10, 2.6	3, 1.2	
Coping strategies			
Insurance (n, %)	31, 7.95	21, 8.37	0.137
Insurance type^a^ (n, %)			
Individual	3, 9.68	1, 4.76	1.078
Community	8, 25.8	2, 9.52	
Corporate	1, 3.23	3, 14.3	
National	19, 61.3	15, 71.4	
Borrow money (n, %)	40, 10.3	19, 7.57	1.319
Lender			
Individual	37, 92.5	18, 94.7	0.125
Social group	1, 2.50	1, 5.26	
Bank	1, 2.50		
Other	1, 2.50		
Sold property (n, %)	39, 10.0	18, 7.2	1.508
Coping costs^b^ mean (SD)			
Borrow money	12.6 (17.7)	8.34 (15.5)	4.25 [−5.41 to 13.9]
Sell property	35.1 (42.7)	31.6 (35.6)	3.47 [−20.3 to 27.3]
Total coping costs	28.9 (38.3)	23.7 (35.3)	5.19 [−11.4 to 21.8]

^a^ Mann–Whitney/Kruskal–Wallis

^b^ column 4 of Coping costs = difference in mean cost [confidence interval]

* *p* < 0.05.

### 3.2 Determinants of patient‐incurred costs

In Table S5, we present the bivariate median regression analysis of determinants of direct, indirect and total costs. The following factors were significantly associated with higher direct medical costs among clinic participants – higher education, owning a small business or having formal employment, and monthly income >$43 (100,000 TZS). Urban residence and higher SES were associated with higher direct costs for both clinic and club participants alike. Higher indirect costs were associated with higher income level and having more visits among all participants, with higher education and formal employment among clinic participants and with primary education and owning a small business among club participants. Conversely, higher total costs were associated with having higher education and higher income level among all participants, with daily labour or formal employment and higher SES among clinic participants and with urban residence and more clinic visits among club participants alone. Sex, age and insurance were not associated with any cost type.

The multivariate median regression model (see Table 5) showed that higher direct costs were associated with higher education and formal employment among clinic participants and with only the highest SES quintile in the club. Increasing years on ART was associated with reduced direct cost among club participants. For indirect costs, higher costs were associated with occupation, that is daily labour, and formal employment among clinic participants, and with owning a small business among club participants. Clinic participants who were more educated and have formal employment had higher total costs. On the other hand, having health insurance was associated with reduced total costs. Higher total costs remained significantly associated with having a formal job, rural residence and more frequent visits among clinic participants.

**Table 5:** Determinants of patient‐incurred costs per year, multivariate median regression

Variables	Direct costs		Indirect costs		Total costs		Difference in total costs
	Coefficient [95% confidence interval]						
	Clinic	Club	Clinic	Club	Clinic	Club	
Sex							
Female	Ref	Ref	Ref	Ref	Ref	Ref	
Male	−0.00 [−3.67 to 3.67]	−0.00 [−0.91 to 0.91]	−1.52 [−6.36 to 3.33]	0.59 [−2.03 to 3.21]	0.72 [−6.29 to 7.73]	2.01 [−1.29 to 5.32]	0.64 [−4.30 to 5.58]
Age							
≤42 years	Ref	Ref	Ref	Ref	Ref	Ref	
>42	−0.00 [−3.43 to 3.43]	0.00 [−0.83 to 0.83]	0.02 [−4.49 to 24.53]	0.06 [−2.32 to 2.45]	3.23 [−3.32 to 9.79]	0.54 [−2.47 to 3.55]	0.87 [−3.66 to 5.40]
Education							
None	Ref	Ref	Ref	Ref	Ref	Ref	
Primary	−0.00 [−3.86 to 3.86]	0.00 [−0.96 to 0.96]	−0.50 [−5.54 to 4.55]	0.11 [−2.67 to 2.90]	0.34 [−7.04 to 9.79]	1.38 [−2.12 to 4.88]	0.65 [−4.55 to 5.85]
≥Secondary	15.6 [7.56 to 23.7]***	0.00 [−2.02 to 2.02]	6.98 [−3.65 to 17.6]	0.26 [−5.75 to 6.28]	20.9 [5.47 to 36.3]**	−0.83 [−8.19 to 6.51]	5.73 [−5.16 to 16.6]
Marital status							
Single	Ref	Ref	Ref	Ref	Ref	Ref	
Married	−3.99 [−8.80 to 0.82]	−0.00 [−1.15 to 1.15]	1.64 [−4.80 to 8.09]	−0.54 [−3.89 to 2.80]	0.65 [−8.53 to 9.84]	−2.87 [−7.07 to 1.32]	−1.67 [−8.02 to 4.67]
Separated/divorced/widow	−1.39 [−6.54 to 3.77]	0.00 [−1.17 to 1.17]	−0.13 [−7.01 to 6.74]	−0.94 [−4.32 to 2.44]	−2.03 [−11.9 to 7.82]	−2.37 [−6.62 to 1.88]	−1.37 [−8.03 to 5.29]
Occupation							
Farming	Ref	Ref	Ref	Ref	Ref	Ref	
Small business	−0.87 [−5.30 to 3.57]	0.00 [−0.94 to 0.94]	2.93 [−2.96 to 8.82]	3.09 [0.38 to 5.79]*	1.75 [−6.73 to 10.2]	3.53 [0.12 to 6.95]*	0.47 [−5.15 to 6.09]
Labourer	0.00 [−5.59 to 5.59]	0.00 [−1.12 to 1.13]	7.54 [0.24 to 14.8]*	1.16 [−2.08 to 4.39]	5.22 [−5.46 to 15.9]	0.66 [−3.42 to 4.75]	0.49 [−6.41 to 7.39]
Formal job	19.9 [7.27 to 32.6]**	−3.47 [−7.36 to 0.42]	22.4 [5.75 to 38.9]**	−1.92 [−17.1 to 13.2]	44.2 [20.0 to 68.5]***	−6.03 [−20.2 to 8.13]	37.5 [19.3 to 55.7]***
Location							
Rural	Ref	Ref	Ref	Ref	Ref	Ref	
Urban	0.87 [−4.44 to 6.17]	−0.00 [−1.23 to 1.23]	−5.91 [−12.9 to 1.06]	−1.21 [−4.78 to 2.35]	−5.03 [−15.2 to 5.10]	−1.88 [−6.35 to 2.58]	−6.68 [−13.0 to −0.31]*
Socio‐economic status (asset index quintile)							
1 (lowest)	Ref	Ref	Ref	Ref	Ref	Ref	Ref
2	0.00 [−4.06 to 4.66]	−0.00 [−1.10 to 1.10]	−0.40 [−6.51 to 5.71]	−1.37 [−4.52 to 1.78]	3.25 [−5.66 to 12.2]	0.57 [−3.42 to 4.55]	1.66 [−5.15 to 8.48]
3	0.00 [−5.29 to 5.29]	0.00 [−1.35 to 1.35]	1.43 [−5.55 to 8.42]	−0.59 [−4.48 to 3.30]	1.77 [−8.34 to 11.9]	−1.12 [−6.03 to 3.79]	2.31 [−5.63 to 10.2]
4	2.60 [−2.32 to 7.52]	0.00 [−1.16 to 1.16]	0.14 [−6.34 to 6.63]	−0.89 [−4.26 to 2.47]	9.01 [−0.40 to 18.4]	0.46 [−3.76 to 4.68]	7.09 [−0.11 to 14.3]
5 (highest)	3.47 [−2.41 to 9.35]	3.47 [2.02 to 4.91]***	1.81 [−5.95 to 9.57]	0.27 [−3.90 to 4.45]	10.3 [−0.90 to 21.6]	3.96 [−1.29 to 9.22]	7.02 [−1.58 to 15.6]
Years on ART							
≤1 year	Ref	Ref	Ref	Ref	Ref	Ref	
1 to 5 years	−2.08 [−9.05 to 4.89]	−6.94 [−10.4 to −3.48]***	–1.19 [−10.3 to 7.89]	–7.66 [−17.6 to 2.27]	–2.36 [−15.7 to 10.9]	–2.27 [−14.8 to 10.3]	–9.46 [−20.1 to 1.19]
>5 years	–2.08 [−9.40 to 5.23]	–6.94 [−10.4 to −3.45]***	–2.68 [−12.2 to 6.85]	–7.17 [−17.2 to 2.85]	–0.58 [−14.6 to 13.4]	−3.39 [−16.1 to 9.27]	–10.5 [−21.4 to 0.50]
Number of visits							
4	Ref	Ref	Ref	Ref	Ref	Ref	
6	0.00 [−6.60 to 6.60]	0.00 [−0.90 to 0.90]	1.50 [−7.09 to 10.1]	2.24 [−0.37 to 4.84]	0.73 [−11.9 to 13.3]	1.76 [−1.52 to 5.04]	6.98 [1.46 to 12.5]**
12	0.00 [−6.96 to 6.96]	0.00 [−1.38 to 1.38]	6.51 [−2.56 to 15.6]	1.66 [−2.31 to 5.64]	2.49 [−10.8 to 15.8]	0.99 [−4.02 to 6.01]	12.2 [5.60 to 18.8]***
Insurance	–0.87 [−7.46 to 5.72]	0.00 [−1.64 to 1.64]	–3.30 [−12.1 to 5.46]	−4.02 [−8.99 to 0.95]	‐13.3 [−25.9 to −0.72]*	−5.87 [−11.8 to 0.10]	‐4.97 [−13.8 to 3.89]

* *p* < 0.05

** *p* < 0.01

*** *p* < 0.001.

## 4 Discussion

We described patient‐incurred costs among clinic‐based and club‐based stable ART patients in North‐west rural Tanzania. These total patient‐incurred costs were significantly higher among clinic participants than those in the clubs. Direct costs were driven by transport costs while income loss was the main contributor to total costs. The incidence of catastrophic expenditure was modest in our study and higher among clinic participants. In addition to the free ART programme, other medical services are provided free by the project sites and likely explains the minimal direct costs incurred by participants on other medications. Most costs were incurred while accessing medical care from other providers between scheduled CTC appointments.

Transportation costs have been identified as the major contributor to direct non‐medical costs among PLHIV in Tanzania as in other parts of Africa [4, 21]. This observation motivated the decentralization of ART services, facilitating the creation of CTC in primary health centres. Nonetheless, the dispersed nature of most rural settings in Tanzania means that PLHIV traverses long distances to get to clinics. Our finding of transport‐related cost reduction especially among rural club participants is consistent with similar past studies on community delivery of ART [32, 33, 34, 35]. Walking, as the most popular means of transport, followed closely by cycling partly explains the reduced costs [4]. Besides walking, a significant decrease in the frequency of service utilization among club participants contributes to the observed cost reduction.

Across studies, income loss during the time spent accessing service is the main driver of indirect costs and consequently total costs [3, 36]. Most participants were informally employed in subsistence farming which makes the opportunity cost for accessing care substantial considering the income levels. The shorter time spent accessing care in the club compared to the clinic, that is travel time and time spent during service, is also reported by similar interventions [32, 37, 38] and directly links the significantly lower income loss seen among club participants compared to the clinic. Minimal disruption of time spent in income‐generating activities resonates across studies on community ART delivery [35, 39, 40]. Similarly, income loss due to illness as revealed in our study is reported in other studies [3, 41].

The frequency of visits is another consideration defining patients’ preferences. Although applying similar recruitment criteria, more visits observed among clinic participants in our study likely reflect the difference in the discretion of clinicians and clients in determining the next clinic visit for clinic and club participants respectively. There were more clients who had been less than two 2 years on ART attending the clinic. These clients tend to have scheduled visits more often which could have contributed to the trend. In our study, stable patients chose the service delivery model that suited their situation, for example despite the cost‐saving benefits of clubs, more men willingly chose to remain in clinics. As such, our results reflect the estimated financial consequences of these choices. Other service delivery models, for example six‐monthly appointments with reduced frequency of clinic visits by increasing months between ART refills may reduce patient‐incurred costs further. However, preference for this model still varies showing the complexity of preferences [42, 43, 44].

Evidence shows that transportation and distance to health facilities contribute to catastrophic expenditure among PLHIV [3, 45]. Adopting a benchmark of >10% of household income to define catastrophic expenditure has been widely used in other studies [3, 46, 47]. Our finding that the proportion of participants experiencing catastrophic expenditure was modest (i.e. <10% of all participants) highlights the low‐income status of this population, to begin with. Additionally, the observation period relatively short, and the comparison was between participants who were stable having minimal clinical symptoms. While catastrophic expenditure is a measure of financial risk, in populations with low incomes the commonly used catastrophic expenditure thresholds resonate poorly. The small proportion of participants reporting coping strategies adopted and no difference between clinic and club reflects the limited options available to participants. Insurance was not available to >90% of study participants and mirrors the status of health insurance coverage in Tanzania which was estimated at 15% (7% – national scheme and 8% – community scheme) in 2014 and 2015 [48, 49].

The trend observed in the distribution of medical expenditures across SES is common in contexts where services are free, such as our study setting. While the poorest in the population appear to have fewer medical costs, this could be interpreted more accurately to reflect the lack of means rather than need. In the face of poverty and the absence of an insurance scheme, options not included in the formal health sector are more likely to be explored.

Our findings should be interpreted in light of some limitations. First, the observational nature of our study design implies that some unobserved factors may have influenced our estimates and confounded our findings. We, however, controlled for basic demographic and socio‐economic factors commonly known to influence patient costs in other observational studies. Second, though we included the money costs of seeking care in between scheduled appointments, we did not include the time costs. Given that most participants report minimal direct cost expenditure due to utilizing the free ART services, we believe our estimates represent the study population fairly. Thirdly, we obtained indirect costs by asking participants to recall expenses incurred in the past six months which posed a risk of recall and measurement biases. The gold standard for estimating income loss, leisure time and its value is through a consumption expenditure questionnaire [50]. In this setting, as in many real‐world situations, it was not possible to conduct such a questionnaire. Our choice of method may have overestimated the value of time if patients attend clubs or clinics when they would not be working. However, these services are mostly available during working hours, so our bias may be limited. Our study compares among stable ART patients alone and results may not necessarily apply to other HIV patients. Our estimates will nonetheless be useful for predicting costs for other patient types who utilize services more frequently. The clinics used in our study being funded by the catholic mission likely influenced the minimal direct costs observed among participants and may therefore not be generalizable to public clinics which are not funded as well. However, in Tanzania, nearly 15% of HIV services are delivered by faith‐based organizations for which this study can be considered representative [51].

## 5 Conclusions

Our study reveals that stable ART patients accessing clinic‐based services incur three times more costs, direct and indirect when compared with club‐based patients. The main cost drivers include transportation and temporary caregivers for direct costs, whereas for indirect costs income loss due to time spent accessing care (both travel and waiting time) was the main driver. Among clinic participants, higher direct costs were associated with higher education and formal employment while among club participants, they were associated with higher SES. Higher indirect costs were associated with formal employment among clinic participants and with higher income levels for both clinic and club participants. Our study, therefore, supports evidence that club‐based care is beneficial to reducing costs and improving access to ART services, especially in rural Tanzania.

## Competing interests

GBG is currently employed by Sanofi Pasteur. Sanofi Pasteur did not provide funding for this study. All other authors declare that they have no competing interests.

## Authors’ contributions

ONE and GBG contributed to the conceptualization and design of the study. ONE conducted the field study and data collection. ONE was responsible for data analysis with guidance from GBG. ONE, LC, DK, SH, DN and TRW were all involved in the interpretation of the results. GBG was responsible for the overall scientific management of the study. ONE wrote the initial draft of the manuscript. All authors contributed to drafts of the manuscript, read and approved the final version.

## Ethics approval

Ethics approval for this research study was obtained from NIMR, that is NIMR/HQ/R.8c/Vol. I/674. Written consent was obtained from individuals who agreed to participate in the study using appropriate forms which had been approved for the same as part of the ethics application.

## Supporting information


**Table S1**. Bivariate analysis showing mean direct, indirect and total costs per year (2019 USD) by participants characteristics irrespective of service delivery model
**Table S2**. Multivariate linear regression showing differences in mean costs per year (2019 USD) between clinics and clubs by participants characteristics
**Table S3**. Oaxaca decomposition
**Table S4**. Annual household income and Medical expenditure in 2019 USD, Opportunity costs per visit and catastrophic expenditure per household
**Table S5**. Bivariate median regression showing factors associated with direct, indirect and total costs per year (2019 USD) incurred by participants
