# Supplementary material for: Patient‐incurred costs in a differentiated service delivery club intervention compared to standard clinical care in Northwest Tanzania
Source: J Int AIDS Soc. 2021 Jun 23;24(6):e25760. doi: 10.1002/jia2.25760 (PMC8222647; doi:10.1002/jia2.25760)
Supplement: Supplementary file 1 — Table S1. Bivariate analysis showing mean direct, indirect and total costs per year (2019 USD) by participants characteristics irrespective of service delivery model Table S2. Multivariate linear regression showing differences in mean costs per year (2019 USD) between clinics and clubs by participants characteristics Table S3. Oaxaca decomposition Table S4. Annual household income and Medical expenditure in 2019 USD, Opportunity costs per visit and catastrophic expenditure per household Table S5. Bivariate median regression showing factors associated with direct, indirect and total costs per year (2019 USD) incurred by participants [file JIA2-24-e25760-s001.docx]

**Supplementary Table S1:** Bivariate analysis showing mean direct, indirect, and total costs per year (2019 USD) by participants characteristics irrespective of service delivery model

|  | **Direct costs** | | **Indirect costs** | | **Total costs** | |
| --- | --- | --- | --- | --- | --- | --- |
|  | ***Mean costs (SD)*** | | | | | |
| **Items** |  | **mean diff [CI]** |  | **mean diff [CI]** |  | **mean diff [CI]** |
| **Sex**   - Female - Male | 7.66 [14.8 ]  10.6 [22.6] | -2.90[-5.80-0.00] | 15.7 [34.8]  16.5 [26.5] | -0.74 [-5.97-4.49] | 18.6[26.0]  26.1[40.3] | **-7.54[-12.7- -2.40]**** |
| **Age**   - ≤42 yrs. - >42 | 8.71 [17.5]  8.71 [18.6] | -0.03[-2.84-2.78] | 16.1 [30.0]  15.9 [33.9] | 0.15 [-4.92-5.23] | 21.7[31.4]  20.9[32.9] | 0.68[-4.31-5.68] |
| **Education**   - None - Primary - ≥Secondary | 5.13 [10.3]  9.47 [19.5]  15.6 [23.6] | Ref  **4.34[1.08-7.59]****  **10.5[3.91-17.1]**** | 13.2 [23.3]  15.8 [32.8]  33.9 [48.8] | Ref  2.59 [-3.27-8.45]  **20.7[8.29-33.2]**** | 17.1[25.6]  21.7[33.0]  36.6[42.7] | Ref  4.57[-1.22-10.4]  **19.5[7.79-31.2]**** |
| **Marital status**   - Single - Married - Separated/Divorced/Widow | 10.4 [17.5]  8.23 [17.9]  8.66 [18.8] | Ref  -2.18[-6.12-1.76]  -1.75[-6.02-2.52] | 15.9 [26.6]  16.7 [29.1]  14.9 [38.9] | Ref  0.85 [-6.35-8.04}  -0.98 [-8.82-6.84] | 24.7[34.1]  22.5[32.5]  17.6[30.3] | Ref  -2.18[-9.18-4.81]  -7.09[-14.7-0.49] |
| **Occupation**   - Farming - Small business - Laborer - Formal Job | 8.84 [19.4]  8.83 [17.3]  5.66 [9.45]  23.8 [33.7] | Ref  -0.01 [-3.56-3.54]  -3.18 [-8.06-.1.70]  **15.0[2.25-27.8]*** | 16.6 [35.0]  15.3 [25.0]  13.8 [18.4]  32.1 [35.2] | Ref  -1.25 [-7.32-4.81]  -2.74 [-11.0-5.52]  -15.5 [-7.57-38.5] | 22.1[35.1]  21.4[30.0]  18.4[20.4]  51.9[61.0] | Ref  -0.62[-7.02-5.77]  -3.68[-12.5-5.11]  **29.8[6.85-52.8]*** |
| **Income level**   - <100,000 - >100,000-300,000 - >300,000 | 7.88 [18.5]  9.29 [14.8]  11.2 [21.4] | Ref  1.41 [-1.85-4.67]  3.29 [-0.85-7.43] | 10.3 [28.1]  19.0 [35.1]  38.5 [32.4] | Ref  **8.69[3.12-14.3]****  **28.2[20.4-35.9]**** | 16.3[32.5]  22.6[22.7]  40.1[38.7] | Ref  **6.36[0.72-11.98]***  **23.8[16.7-30.9]**** |
| **Location**   - Rural - Urban | 9.11 [19.8]  7.34 [7.34] | Ref  1.77 [-1.63-5.17] | 17.4 [34.1]  10.4 [20.5] | Ref  **7.02[0.74-13.3]*** | 23.4[35.0]  14.0[17.0] | Ref  **9.36[3.34-15.4]**** |
| **Socio-economic status**   - 1 (Lowest) - 2 - 3 - 4 - 5 (Highest) | 5.45[14.6]  8.00[14.1]  10.0[27.1]  9.99[18.6]  11.9[17.9] | **Ref**  2.54[-1.53-6.62]  4.57[-0.16-9.31]  **4.53[0.37-8.69]***  **6.42[2.29-10.5]**** | 13.7 [30.7]  14.3 [29.4]  13.9 [19.5]  18.5 [38.4]  20.2 [35.7] | Ref  0.55 [-6.81-7.92]  0.21 [-8.39-8.81]  4.79 [-2.71-12.3]  6.49 [-1.08-14.1] | 16.3[27.9]  19.8[27.9]  22.7[38.6]  23.6[33.9]  26.9[34.9] | Ref  3.44[-3.82-10.7]  6.37[-2.01-14.8]  7.26[-0.16-14.7]  **10.5[3.14-17.9]**** |
| **Years on ART**   - ≤ 1 year - 1 – 5 years - > 5 years | 8.66 [13.0]  8.58 [17.8]  8.66 [18.5] | Ref  -0.08 [-7.09-6.94]  0.00 [-7.14-7.14] | 19.9 [34.8]  14.4 [26.6]  17.1 [35.4] | Ref  -5.52[-17.6-6.52]  -2.82 [-15.1-9.42] | 26.7[34.2]  20.6[29.3]  21.7[35.3] | Ref  -6.11[-18.6-6.39]  -4.96[-17.7-7.76] |
| **Number of visits**   - 4 - 6 - 12 | 4.93 [12.7]  9.07 [17.6]  12.1 [23.1] | Ref  **4.13[0.72-7.52]***  **7.15[3.04-11.3]**** | 6.84 [17.9]  17.1 [31.5]  23.8 [41.6] | Ref  **10.2[4.14-16.3]****  **16.9[9.58-24.3]**** | 10.4[17.7]  23.2[34.5]  28.8[35.7] | Ref  **12.8[6.83-18.8**]**  **18.4[11.2-25.6]**** |
| **Insurance**   - Yes - No | 9.36 [16.3]  8.75 [18.3] | Ref  0.67 [-4.48-5.81] | 22.7 [37.8]  15.3 [31.3] | Ref  7.31 [-2.02-16.6] | 27.9[36.6]  20.7[31.7] | Ref  7.19[-1.95-16.3] |
| **Club**   - Clinic - Club | 11.6 [21.0]  4.17 [10.7] | Ref  **7.49[4.67-10.3]**** | 20.9 [36.7]  8.23 [20.1] | **Ref**  **12.6[7.53-17.8]**** | 32.2[45.7]  12.4[25.2] | Ref  19.7[13.4 – 26.1]** |

Table S1 highlights the bivariate associations of direct, indirect and total costs incurred per year by participant characteristics. The costs presented are the mean annual costs with standard deviation and the difference in mean annual costs with confidence intervals

**Legend:**

**bold** = coefficients with significant p values; * = p values < 0.05 ; ** = p values < 0.01; ***=p values <0.001

Mean diff = Difference in mean costs

**Supplementary Table S2:** Multivariate Linear regression showing difference in mean costs per year (2019 USD) between clinics and clubs by participants characteristics

| **Items** | **Direct costs** | | **Indirect costs** | | **Total costs** | |
| --- | --- | --- | --- | --- | --- | --- |
|  | **Mean diff**  **(SD)** | **Coeff [CI]** | **Mean diff**  **(SD)** | **Coeff [CI]** | **Mean diff**  **(SD)** | **Coeff [CI]** |
| **Sex**   - Female - Male | 6.84 (29.1)  8.14 (47.2) | 0.22[-7.81-8.24] | 13.9 (69.5)  10.4 (55.1) | 1.71[-10.3-13.7] | 20.3 (78.2)  18.4 (85.3) | 1.73[-14.4-17.9] |
| **Age**   - ≤42 yrs. - >42 | 7.68 (37.0)  7.69 (36.6) | 0.95[-3.90-5.80] | 13.6 (63.4)  12.3 (67.1) | 8.47[-0.58-17.5] | 20.6 (79.0)  20.0 (83.8) | Ref  9.36[-1.87-20.6] |
| **Education**   - None - Primary - ≥Secondary | 4.15 (20.8)  7.75 (39.0)  19.6 (47.5) | -2.79[-7.69-2.11]  **-17.3[-31.4- -3.13]*** | 14.6 (46.5)  11.6 (65.8)  13.9 (107.4) | 2.78[-6.71-12.3]  8.24[-38.3-54.8] | 18.5 (54.2)  19.0 (83.6)  32.9 (125.4) | Ref  -0.18[-11.5-11.2]  -9.05[-61.6-43.5] |
| **Marital status**   - Single - Married - Separated/Divorced/Widow | 9.77 (34.4)  8.28 (36.5)  5.33 (37.5) | 7.23[-0.52-15.0]  7.99[-0.20-16.2] | 8.88 (53.6)  13.7 (59.6)  12.7 (77.5) | -1.71[-13.3-9.88]  -1.96[-15.4-11.5] | 18.0 (70.3)  21.4 (77.1)  18.2 (91.4) | Ref  5.12[-11.4-21.7]  6.18[-11.6-23.9] |
| **Occupation**   - Farming - Small business - Laborer - Formal Job | 6.70 (42.5)  7.91 (33.9)  6.59 (17.9)  29.5 (76.4) | 0.76[-7.40-8.93]  1.66[-4.14-7.47]  -18.2[-42.9-6.58] | 14.0 (76.2)  11.7 (48.9)  15.2 (33.7)  34.2 | 4.54[-4.39-13.5]  -3.33[-13.6-6.98]  -32.5[-65.4-0.39] | 20.6 (95.4)  19.3 (68.1)  21.8 (37.2)  62.0 | Ref  3.55[-9.05-16.2]  -2.87[-15.3-9.34]  **-53.2[-99.9- -6.55]*** |
| **Location**   - Rural - Urban | 7.80 (41.2)  6.60 (18.5) | 5.69[-1.34-12.7] | 13.2 (71.2)  7.52 (41.0) | 10.2[-0.99-21.4] | 20.5 (89.0)  13.6 (50.9) | Ref  **15.4[0.82-29.9]*** |
| **Socio-economic status**   - 1 (Lowest) - 2 - 3 - 4 - 5 (Highest) | 5.15 (29.7)  2.82 (29.4)  8.07 (57.6)  10.0 (36.2)  13.2 (33.8) | 3.01[-3.67-9.69]  -0.49[-14.4-13.4]  -1.87[-9.24-5.50]  0.77[-6.64-8.17] | 10.7 (62.6)  10.7 (61.2)  8.02 (41.2)  20.4 (74.8)  14.5 (71.3) | -0.57[-13.2-12.0]  -0.70[-12.7-11.3]  -7.85[-20.4-4.73]  -3.22[-18.5-12.1] | 15.7 (75.1)  13.5 (70.9)  16.2 (82.8)  29.0 (86.6)  27.2 (88.7) | Ref  3.23[-11.8-18.3]  -1.48[-22.8-19.8]  -9.39[-25.5-6.75]  -2.03[-20.9-16.9] |
| **Years on ART**   - ≤ 1 year - 1 – 5 years - > 5 years | 1.51 (37.4)  7.07 (36.1)  7.74 (36.6) | -1.32[-8.68-6.04]  -3.61[-11.9-4.65] | 17.9 (98.2)  10.3 (54.3)  14.1 (70.0) | 2.70[-16.00-21.4]  -0.24[-20.7-20.2] | 19.5 (101.6)  16.6 (68.8)  22.1 (87.8) | Ref  1.71[-16.3-19.7]  -3.79[-24.2-16.6] |
| **Number of visits**   - 4 - 6 - 12 | 3.30 (34.6)  7.28 (38.7)  9.39 (58.8) | -4.33[-10.8-2.15]  -6.67[-15.0-1.67] | 1.32 (48.4)  9.45 (71.0)  16.8 (107.6) | **-9.09[-17.9-0.30]***  **-15.5[-29.3- -1.61]*** | 4.45 (63.4)  16.2 (88.6)  26.7 (132.8) | Ref  **-13.2[-24.6- -1.71]***  **-21.4[-37.6- -5.25]**** |
| **Insurance**   - Yes - No | 11.9 (31.4)  7.11 (36.8) | 0.22[-8.61-9.06] | 7.80 (77.9)  13.2 (63.2) | -11.7[-42.4-18.9] | 19.2 (90.4)  20.0 (79.7) | Ref  -10.6[-44.7-23.5] |

Table S2 highlights the multivariate association estimates of the difference in mean costs (i.e. direct, indirect and total costs incurred per year) between clinics and clubs by participant characteristics. The estimates presented are the coefficients with confidence intervals showing the strength of association between each cost type and each participant chahracteristic.

**Legend:**

**bold** = coefficients with significant p values; * = p values < 0.05 ; ** = p values < 0.01; ***=p values <0.001

**Supplementary Table S3:** OAXACA decomposition showing the percentage of variations in Total costs per year (in 2019 USD) attributable to participants characteristics

| **Summary of decomposition results:** | | | | | |
| --- | --- | --- | --- | --- | --- |
| Variation due to participants characteristics | 0,719 | 4,8% | % of variation in total costs attributable to differences in participant characteristics included in the regression model across intervention groups | | |
| Variation due to coefficients | 12,427 | 83,4% | % of variation in total costs attributable to differences unexplained by differences in participant characteristics included in the regression model e.g. due to effect of the intervention | | |
| Variation due to interaction | 1,751 | 11,8% | % of variation in total costs attributable to an interaction of the effect of participants characteristics and intervention effect | | |
|  | | | | | |
|  | 0 | 1 | 0,5 | 0,636 | * |
| Unexplained variation | 14,178 | 12,427 | 13,302 | 13,064 | 9,303 |
| Explained variation | 0,719 | 2,47 | 1,595 | 1,834 | 5,594 |
| % unexplained | 95,2 | 83,4 | 89,3 | 87,7 | 62,4 |
| % explained | 4,8 | 16,6 | 10,7 | 12,3 | 37,6 |

Table S3 shows the results of the OAXACA decomposition which highlights the percentage of variation seen in total costs incurred by participants per year which is attributable to participants characteristics or to the service delivery model. The estimates presented in the second part of the table highlights differences in percentage variation explained or unexplained by participants characteristics. Column ‘0’ shows the Oaxaca decomposition which places the interaction in the unexplained part, while in column ‘1’, the interaction is placed in the explained part. Columns ‘0.5’ and ‘0.636’ shows the results of other decomposition types namely, ‘Cotton’ and ‘Reimer’ decompositions while column ‘*’ highlights the ‘Neumark’s’ decompossiton, which uses coefficients from the pooled regression. Which ever decomposition is considered, participants characteristics explain a smaller percentage of the variation seen in mean total costs.

**Supplementary Table S4:** Annual household income and Medical expenditure in 2019 USD, Opportunity costs per visit and catastrophic expenditure per household

| **Household income per year** *mean(SD)* | Clinic (N=390) | Club (N=251) | p-value |
| --- | --- | --- | --- |
| - Main income - Additional income - Total household income | 873.2 (2285.6)  505.5 (603.3)  1069.9 (2402.1) | 698.0 (976.9)  378.5 (508.1)  816.6 (1001.1) | 175.2[-124.4-474.7]  127.0[-58.2-312.2]  253.4[-60.6-567.4] |
| **Medical care expenditure per year** |  |  |  |
| - Direct cost - Indirect cost - Total expenditure | 11.7 (21.0)  20.9 (36.7)  31.8 (45.4) | 4.17 (10.7)  8.23 (20.1)  11.9 (24.5) | 7.49 [4.67 – 10.30]***  12.6 [7.53 – 17.8]***  19.7 [13.4 – 26.1]*** |
| **Opportunity costs**   - Income loss per visit - Income loss per visit ^(Drummond) - Difference in income loss | 1.68 (2.57)  1.65 (2.51)  0.02 (0.53) | 0.81 (1.43)  0.76 (1.28)  0.04 (0.54) | 0.87[0.51-1.23]***  0.89[0.55-1.23]***  -0.02[-0.01-0.07] |
| **Catastrophic expenditure** *(n, %)* |  |  |  |
| Proportion with medical expenditure   - >5% of household income - >10% of household income - >20% of household income | 92, 23.6  42, 10.8  23, 5.90 | 23, 9.16  13, 5.18  5, 1.9 | 21.589***  6.083  5.576* |

**Table S4** shows household income and medical expenditure per year and opportunity costs, highlighting the differences between clinic and clubs. The percentage of households experiencing catastrophic expenditure when using the benchmarks of 5%, 10% and 20% of household income are also presented showing the difference between clinic and clubs. All incomes and costs are presented in USD.

**Legend:** ^Drummond method assigns the minimum wage to participants who are not employed. We assigned a monthly income of 100,000TZS to all participants who are unemployed to capture the values for activities forgone such are Family care/Child care. The value per productive hour was calculated on that basis and used to assess income loss per visit; p values *< 0.05 ; ** = p values < 0.01; ***=p values <0.001

**Supplementary Table S5:** Bivariate median regression showing factors associated with direct, indirect, and total costs per year (2019 USD) incurred by participants

|  | **Direct costs** | | **Indirect costs** | | **Total costs** | |
| --- | --- | --- | --- | --- | --- | --- |
|  | ***Coefficient (95% CI)*** | | | | | |
| **Items** | **Clinic** | **Club** | **Clinic** | **Club** | **Clinic** | **Club** |
| **Sex**   - Female - Male | Ref  0 [-3.97-3.97] | Ref  0[-0.92-0.92] | Ref  0.49 [-2.69-3.67] | Ref  0.76[-0.77-2.30] | Ref  2.13[-3.80-8.06] | Ref  -0.06[-3.01-3.14] |
| **Age**   - ≤42 yrs. - >42 | Ref  0 [-3.60-3.60] | Ref  0 [-0.83-0.83] | Ref  -2.36 [-5.24-0.52] | Ref  -0.81 [-2.31-0.68] | Ref  3.56[-2.20-9.33] | Ref  -0.87[-3.69-1.96] |
| **Education**   - None - Primary - ≥Secondary | Ref  0 [-3.38-3.38]]  **15.6[9.08-22.1]**** | Ref  0 [-0.97-0.97]  0 [-2.02-2.02] | Ref  -0.97 [-4.85-2.90]  **10.6[3.07-18.1]**** | Ref  **2.17[0.39-3.94]***  0.32 [-3.58-4.23] | Ref  -3.34[-3.47-10.1]  **22.9[9.72-36.0]*** | Ref  **4.31[1.17-7.44]****  -0.34[-7.24-6.55] |
| **Marital status**   - Single - Married - Separated/Divorced/Widow | Ref  **-6.94[-10.3--3.56]****  -0.69 [-4.44-3.05] | Ref  0 [-0.68-0.68]  0 [-0.71-0.71] | Ref  3.12 [-0.61-6.85]  0 [-4.14-4.14] | Ref  0.35 [-1.67-2.37]  -0.03 [-2.35-1.87] | Ref  -3.55[-11.6-4.49]  -5.27[-14.2-3.65] | Ref  -1.52[-5.79-2.74]  -1.16[-5.63-3.30] |
| **Occupation**   - Farming - Small business - Laborer - Formal Job | Ref  **5.20[-0.41-9.99]***  2.60[-4.14-9.35]  **26.0[10.9-41.1]**** | Ref  0 [-0.75-0.75]  0 [-0.99-0.99]  0 [-3.37-3.37] | Ref  3.65 [-0.22-7.51]  **5.89[0.45-11.3]***  **22.0[9.81-34.2]**** | Ref  **3.42[2.05-4.79]****  1.06 [-0.74-2.85]  -1.09 [-7.22-5.02] | Ref  5.09[-1.90-12.1]  **11.1[-1.23-20.9]***  **60.4[38.3-82.5]**** | Ref  **5.26[2.21-8.32]****  0.45[-3.55-4.46]  -2.11[-15.8-11.5] |
| **#Income level** *monthly*   - <100,000 - >100,000-300,000 - >300,000 | Ref  **5.20[1.26-9.15]***  **9.11[3.87-14.3]**** | Ref  0 [-0.31-0.31]  0 [-0.37-0.37] | Ref  **9.49[6.64-12.3]****  **32.7[28.9-36.5]**** | Ref  **3.49[2.18-4.79]****  **7.35[5.80-8.89]**** | Ref  **15.6[9.96-21.2]****  **41.8[34.1-49.3]**** | Ref  **3.82[1.63-6.01]****  **9.09[6.49-11.7]**** |
| **Years on ART**   - ≤ 1 year - 1 – 5 years - > 5 years | Ref  -7.28 [-15.6-1.07]  -7.28 [-15.9-1.33] | Ref  **-6.94[-10.1- -3.81]****  **-6.94[-10.1- -3.79]**** | Ref  0.95 [-5.19-7.09]  1.44 [-4.90-7.77] | Ref  -2.66 [-8.56-3.23]  -3.29 [-9.22-2.62] | Ref  0.69[-11.4-12.8]  2.13[-10.3-14.6] | Ref  -2.07[-13.3-9.12]  -2.67[-13.9-8.57] |
| **Location**   - Rural - Urban | Ref  **10.4[5.25-15.6]**** | Ref  **3.47[2.73-4.21]**** | Ref  -0.38 [-4.82-4.04] | Ref  0.81 [-0.76-2.39] | Ref  -0.57[-8.71-7.56] | Ref  **3.68[0.99-6.36]*** |
| **Number of visits**   - 4 - 6 - 12 | Ref  0 [-7.89-7.89]  0 [-8.31-8.31] | Ref  0 [-0.68-0.68]  0 [-1.08-1.08] | Ref  2.67 [-3.73-9.06]  **7.52[0.78-14.3]*** | Ref  1.75*[0.17-3.32]  2.83*[0.32-5.34] | Ref  2.84[-9.97-15.6]  7.25[-6.24-20.7] | Ref  **4.30[1.51-7.09]****  **4.28[0.05-8.72]*** |
| **Insurance** | **10.4[2.92-17.9]**** | 0 [-1.66-1.66] | -0.15[-5.74-5.44] | 2.49 [-0.05-5.04] | 7.81[-2.45-18.1] | 1.56[-3.49-6.61] |
| **Socio-economic status**  **(**Asset index quintile)   - 1 (Lowest) - 2 - 3 - 4 - 5 (Highest) | Ref  0 [-3.91-3.91]  0 [-4.46-4.46]  **7.28[3.14-11.4]****  **10.4[6.33-14.5]**** | Ref  0 [-0.68-0.68]  0 [-0.82-0.82]  0 [-0.65-0.65]  **3.47[2.81-4.13]**** | Ref  -0.44[-5.17-4.29]  -0.15 [-5.54-5.25]  2.55 [-2.47-7.58]  3.56 [-1.38-8.49] | Ref  -1.44 [-3.78-0.89]  -0.45 [-3.27-2.36]  -0.03 [-2.28-2.22]  0.69 [-1.57-2.95] | Ref  1.76[-5.35-8.87]  2.63[-5.49-10.7]  **12.5[4.97-20.1]****  **13.4[6.01-20.9]**** | Ref  -1.81[-5.81-2.20]  -1.87[-6.70-2.95]  0.28[-3.57-4.14]  2.49[-1.38-6.37] |

**Table S5** presents the results of the bivariate analysis showing associations between participants characteristics (in clinics and clubs) and median direct, indirect and total costs in USD.

**Legend:**

**# =** <100,000 = $43.1; 100,000 – 300,000 = $43.1 – 130.1; >300,000 = > $130.1

**bold** = coefficients with significant p values; * = p values < 0.05 ; ** = p values < 0.01; ***=p values <0.001
